# P-2164. Clinically Significant Cytomegalovirus Infection (csCMVi) After Matched Donor Allogeneic HSCT Using Post-Transplant Cyclophosphamide: A Systematic Review and Meta-Analysis

**DOI:** 10.1093/ofid/ofaf695.2327

**Published:** 2026-01-11

**Authors:** Varshini Thiruvadi, Shweta Kapur, Abul Hasan Shadali, Saba Asif, Pranatharthi Chandrasekar, Lea M Monday, Vishakh C Keri

**Affiliations:** University of Illinois College of Medicine, Peoria, Peoria, IL; Wayne state University/ DMC, Detroit, Michigan; Apollo Hospital, Greams Road, Chennai, India, Chennai, Tamil Nadu, India; Trinity Health Oakland/ Wayne State University, Pontiac, Michigan; wayne state university, detroit, MI; Wayne state University School of Medicine, Detroit, Michigan; Wayne State University, Detroit, MI

## Abstract

**Background:**

Post-transplant cyclophosphamide (PTCy) was initially used for graft-versus-host disease (GVHD) prophylaxis in haploidentical allogeneic hematopoietic stem cell transplantation (allo-HSCT). This strategy is now expanding into matched related and unrelated donor allo-HSCT. However, the risk of clinically significant cytomegalovirus infection (csCMVi) including reactivation and end-organ disease is poorly defined in this population. We aimed to systematically evaluate the cumulative incidence of csCMVi in this subgroup.

**Figure:**
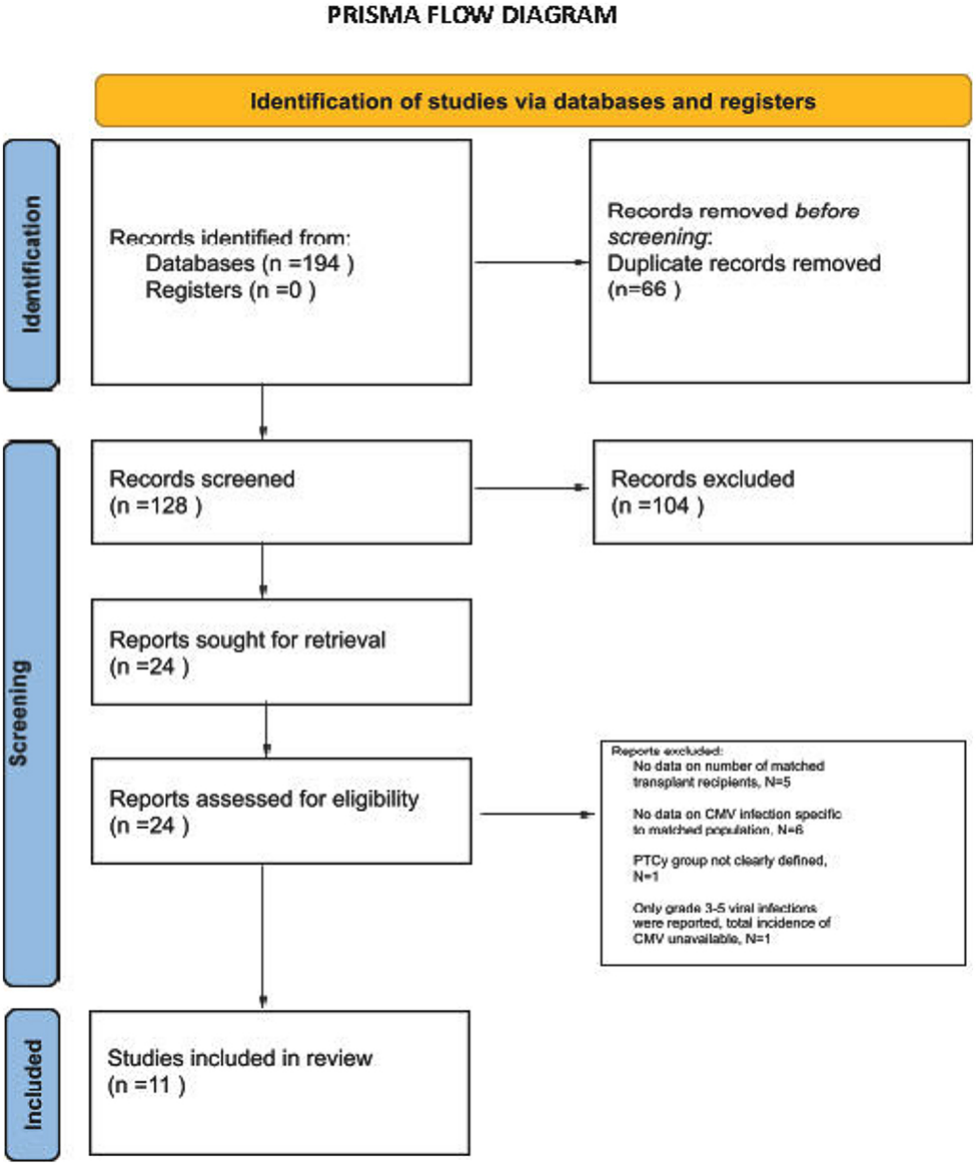
PRISMA FLOW DIAGRAM

**Figure:**
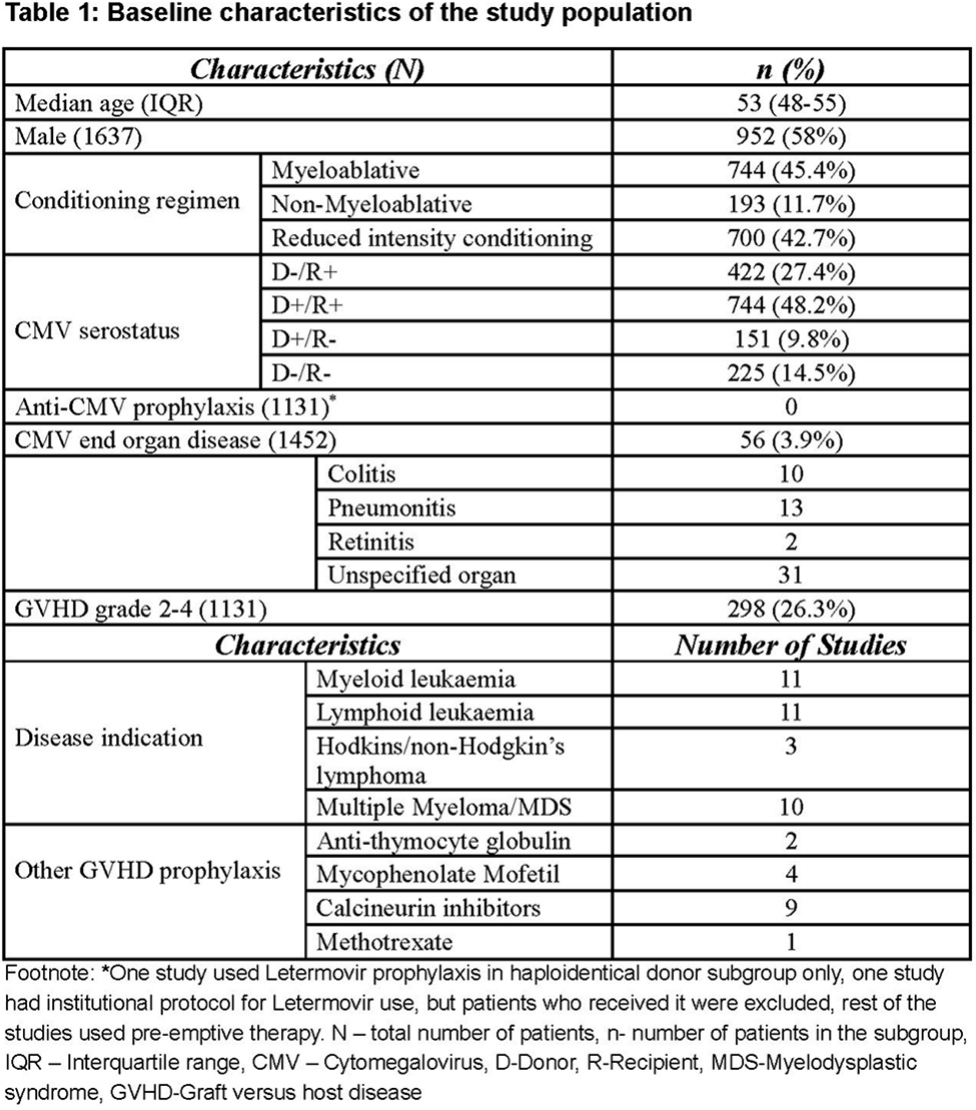
Baseline characteristics of the study population

**Methods:**

A systematic review was conducted according to PRISMA guidelines and registered in PROSPERO (ID: 1043133). PubMed, Embase, Scopus, and Cochrane CENTRAL were searched for studies of csCMVi incidence in adult patients with hematologic malignancies undergoing matched donor allo-HSCT with PTCy-based GVHD prophylaxis. A proportion meta-analysis was performed using a random-effects model with Freeman-Tukey double arcsine transformation. Heterogeneity was assessed with I² and Tau²; publication bias was evaluated by funnel plot symmetry.

**Figure:**
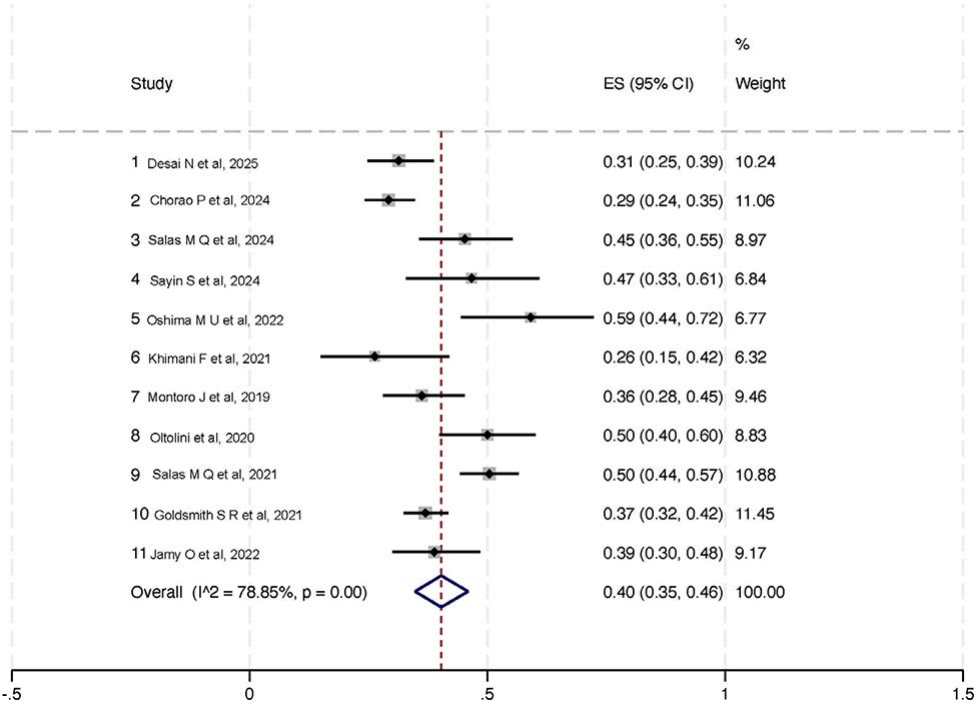
Forest plot

**Figure:**
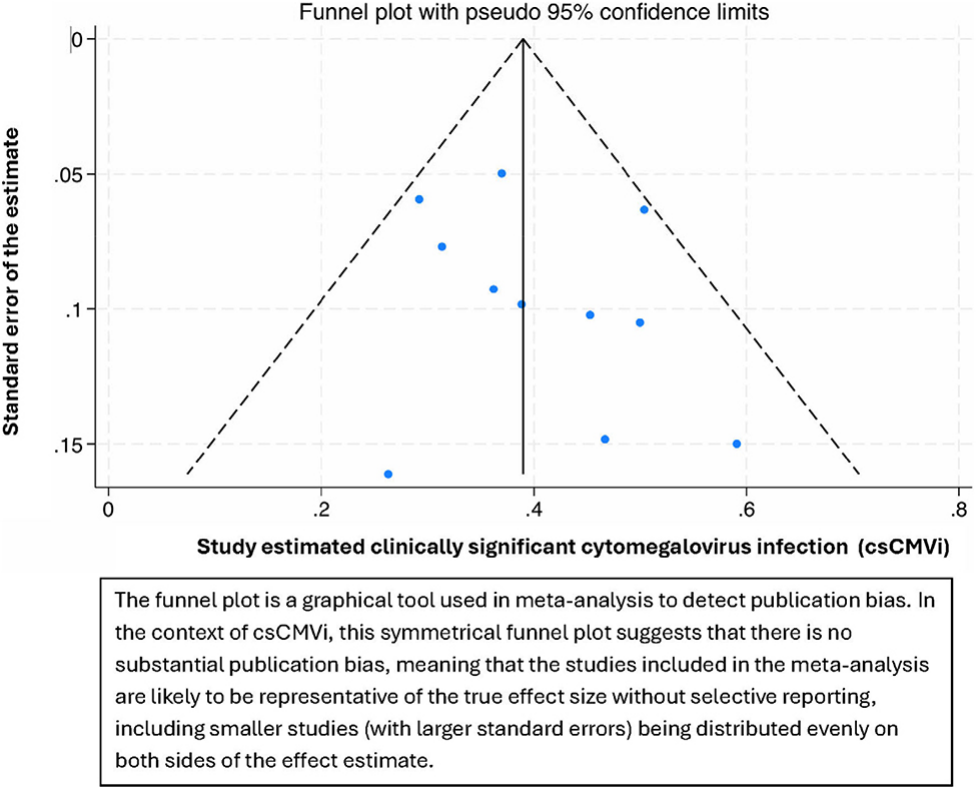
Publication bias assessed by Funnel Plot

**Results:**

Eleven studies comprising 1,637 patients, with study periods ranging from 2007 to 2023, were included. The most common CMV serostatus was D+/R+ (48.2%), followed by D-/R+ (27.4%). None of the studies reported use of anti-CMV prophylaxis. The pooled cumulative incidence of csCMVi was 40% (95% CI: 35–46%). Heterogeneity was substantial (I² = 78.85%, Tau² = 0.03),

and a random-effects model was used. The funnel plot was symmetrical and did not suggest substantial publication bias. CMV end-organ disease occurred in 3.9% of patients; grade 2–4 acute GVHD occurred in 26.3%.

**Conclusion:**

Clinically significant CMV infection remains a substantial burden among matched donor allo-HSCT recipients receiving PTCy, with a pooled incidence of 40%. Our findings highlight a critical evidence gap as PTCy expands into matched donor transplantation. The consistent absence of anti-CMV prophylaxis such as Letermovir underscores the need for standardized prevention protocols. Notably, data on CMV incidence in patients receiving prophylaxis are lacking, warranting further studies.

**Disclosures:**

All Authors: No reported disclosures

